# Detection by super-resolution microscopy of viral proteins inside bloodborne extracellular vesicles

**DOI:** 10.20517/evcna.2023.46

**Published:** 2023-11-01

**Authors:** Rakesh K. Singh, Mark F. Santos, Charles Herndon, Brandon A. Gieler, Isaac Lee, Jiahui Chen, Aurelio Lorico

**Affiliations:** Department of Basic Sciences, Touro University Nevada, Henderson, NV 89014, USA.; ^#^Authors contributed equally.

**Keywords:** Extracellular vesicles, super-resolution microscopy, permeabilization, intravesicular cargo, SARS-CoV-2, spike, COVID-19

## Abstract

**Aim:**

Extracellular vesicles (EVs) are small particles released by all cells, including virally infected cells, into the extracellular space. They play a role in various cellular processes, including intercellular communication, signaling, and immunity, and carry several biomolecules like proteins, lipids, and nucleic acids that can modulate cellular functions mostly by releasing their cargo inside the target cells via the endocytic pathway. One of the most exciting aspects of EV physiology is its potential in liquid biopsy as a diagnostic and prognostic marker. However, due to their extremely small size and lack of a molecular approach to examine intravesicular content or cargo, we cannot fully utilize their potential in healthcare.

**Methods:**

Here, we present a novel approach that allows examining bloodborne EVs at a single-particle level with the ability to examine their cargo without disrupting structural integrity. Our technique utilizes super-resolution microscopy and a unique permeabilization process that maintains structural integrity while facilitating the examination of EV cargo. We used a mild-detergent-based permeabilization buffer that protects the integrity of EVs, minimizes background, and improves detection.

**Results:**

Utilizing this approach, we were able to recognize viral proteins of SARS-CoV-2 virus in COVID-19 patients, including spike and nucleocapsid. Surprisingly, we found an almost equal amount of spike protein inside and on the surface of bloodborne EVs. This would have proven difficult to determine using other conventional methods.

**Conclusion:**

To summarize, we have developed an easy-to-perform, sensitive, and highly efficient method that offers a mechanism to examine bloodborne EV cargo without disrupting their structural integrity.

## INTRODUCTION

Extracellular vesicles (EVs) are small particles released by all cells, including infected cells, into the extracellular space. EVs can travel to other cells and deliver their cargo, which can affect the target cells. They are involved in various functions, including intercellular communication, signaling, and immunity. EVs include exosomes, derived from multi-vesicular bodies and ectosomes, shed by the plasma membrane^[1]^. The former range in size between 30 and 150 nm and have some size overlap with ectosomes, although ectosomes may be larger than 150 nm.

The similar size, composition, biogenesis, intracellular transport, and egress analogies between exosomes and many viruses^[2-7]^ are the basis for the Trojan exosome hypothesis^[4]^. In addition to this substantial overlap, viral proteins and nucleic acids are also found in EVs released from infected cells, such as HIV-1 Nef protein^[8]^ and transactivation response element (TAR) RNA^[9]^ and viral spike or envelope glycoproteins on their surface from a variety of viruses^[10-12]^. EVs from virally infected cells can also stimulate T cells either directly by presenting to T cells peptides on major histocompatibility complex (MHC) or indirectly through uptake by dendritic cells (DC) and subsequent processing of antigens for presentation, although the former has weaker cross-presentation ability^[13-17]^. Recent reports have also confirmed the involvement of EVs derived from plasmacytoid DCs in transferring antigens to tissue-resident DCs to activate CD8^+^ T cells^[18]^. Plasmacytoid DCs reside in peripheral blood and have an important role in antiviral immunity, mainly through the release of type I interferon^[19]^. Finally, a recent study has shown that some EVs derived from platelets can elicit an immune response through antigen presentation, wherein a peptide-MHC class I complex and co-stimulatory molecules interactions occur at the immune synapse with CD8^+^ T cells^[20]^. EVs also contain functional proteasomes that can enable peptide generation for antigen presentation^[20]^.

Recent reports have identified viral proteins carried by EVs, including spike and nucleocapsid protein components of severe acute respiratory syndrome coronavirus 2 (SARS-CoV-2)^[21-23]^, a virus that has caused the global COVID-19 pandemic with clinical symptoms ranging from asymptomatic infection to respiratory failure and death^[24]^. Before the role of EVs in COVID-19 or other viral diseases and/or as diagnostic/prognostic markers for a wide variety of disease states can be established, numerous challenges must be addressed. The first and foremost is the lack of a reliable and reproducible technique to analyze the cargo and origin of single EVs circulating in human blood. One potential approach is super-resolution microscopy, which enables the imaging of individual EVs. However, the methods described so far have been mostly limited to the analysis of surface proteins. When performed carefully, permeabilization allows for the entry of probes or antibodies without destroying the physical characteristics of EVs. An efficient permeabilization protocol, together with super-resolution microscopy, would provide important information about the cargo inside the EVs and about the cellular origin of each single EV, leading to significant progress in the clinical diagnosis of diseases via liquid biopsy. In comparison, previous studies aimed at identifying SARS-CoV-2 markers in blood plasma EVs have utilized mass spectrometry and nanoscale flow cytometry^[22]^, have developed methods to permeabilize EVs isolated from cells in culture^[25]^, or performed immunoblotting on EV lysates^[26]^. The latter process requires an extensive amount of EVs, and analysis may not be possible with small blood samples. Additionally, these methods do not enable the determination of the size, shape, or source of circulating EVs in the blood. Here, we set out to establish a reproducible and highly efficient methodology to examine the intravesicular content of EVs isolated from the blood plasma of healthy volunteers and SARS-CoV2-infected patients.

## MATERIALS AND METHODS

### Cell culture

FEMX-I cells were originally derived from a lymph node metastasis of a patient with malignant melanoma^[27]^ and were authenticated by morphology and proteomics^[28]^. They were cultured as described in our previous publications^[27,28]^. In brief, cells were grown in RPMI-1640 medium (catalog number (#) 10-041-CV, Corning Inc.) supplemented with 10% fetal bovine serum (FBS, #26140079, Thermo Fisher Scientific), 2 mM L-glutamine (#25030081, Thermo Fisher Scientific), 100 U/mL penicillin and 100 μg/mL streptomycin (#15140122, Thermo Fisher Scientific), and incubated at 37 °C in a 5% CO_2_ humidified incubator. They were regularly verified for absence of mycoplasma contamination by either their staining with 4',6-diamidino-2-phenylindole (DAPI; #D9542, Sigma-Aldrich) and visualization under a fluorescent microscope or polymerase chain reaction using the MycoSEQ™ Mycoplasma Detection Kit (#4460626, Thermo Fisher Scientific), according to the manufacturer’s protocol.

### Transfection

Using Lipofectamine 3000 (#L3000008, Thermo Fisher Scientific), FEMX-I cells were transfected with the pCMV6-AN-mGFP mammalian expression vector (#PS100048, OriGene) to express green fluorescent protein (GFP). Transfected cells were selected by introducing 400 µg/mL of Geneticin (G418 Sulfate, #10131027, Thermo Fisher Scientific) into the culture medium. Selection antibiotics were removed from the medium at least one week before experiments.

### Blood collection from COVID-19 patients

COVID-19 positive patients, tested with FDA-approved self-administered COVID-19 kits, were enrolled in the study after approval by the Touro University Nevada Institutional Review Board for Human Subjects Research (TUNIRB). Individuals signed informed and written consent for a protocol approved by TUNIRB (000132). Approximately 10 mL of blood was drawn by venipuncture at the TUN Clinic.

### Isolation and characterization of EVs

EVs were prepared from parental or GFP-transfected FEMX-I cells (250,000 cells). Herein, cells grown on 6-well plates pre-coated with 20 µg/mL poly(2-hydroxyethyl methacrylate) (#P3932, Sigma-Aldrich) to prevent their attachment, as described in^[27]^, were cultured in serum-free medium supplemented with 2% B-27 supplement (#17504044, Thermo Fisher Scientific). After 72 h, EVs were enriched by serial centrifugation at low speed (300 and 1,200 × *g*) followed by centrifugation at 10,000 × *g* for 30 min at 4 °C. The resulting supernatant was centrifuged at 200,000 × *g* for 60 min at 4 °C. The pellet was resuspended in 200 µl PBS. For EVs isolated from blood plasma, blood collected on K2 EDTA vacutainer tubes (#BD-367863, VWR) was centrifuged at 2,000 × *g* for 10 min at 4 °C to separate the plasma layer. Plasma was then diluted with PBS at a 1:1 ratio and processed as described above to isolate EVs. Nanoparticle tracking analysis (NTA) with ZetaView (Particle Metrix GmbH) was utilized to determine the size and concentrations of EVs. The following parameters were used: 488 nm laser in scatter mode, video duration of 2 seconds at 30 frames per second for 11 positions along the z-axis of the analysis window, camera gain of 10, and trace length of 15. EVs were characterized for the presence (CD9, CD81, CD63, Alix, and Syntenin) or absence (calnexin) of particular proteins by immunoblotting (see below) according to the guidelines of the International Society for Extracellular Vesicles (MISEV2018)^[29]^.

### Immunoblotting

Cells were lysed with 1% Triton X-100 (#X100, Sigma-Aldrich), 100 mM NaCl, 50 mM Tris-HCl, pH 7.5 in the presence of Protease Inhibitors (#539134, Sigma-Aldrich) followed by incubation on ice for 30 min. Samples were prepared and proteins separated by SDS-PAGE and transferred to a nitrocellulose membrane as described in^[30]^. Membranes were incubated in the blocking buffer [PBS containing 1% bovine serum albumin (BSA, #001-000-161, Jackson ImmunoResearch Laboratories Inc.)] for 60 min at room temperature (RT), and then probed with anti-Alix (#2171, Cell Signaling Technology), anti-Calnexin (#MA3-027, Thermo Fisher Scientific), anti-CD63 (#10-628-D, Thermo Fisher Scientific), anti-CD9 (#sc-20048, Santa Cruz Biotechnology), anti-CD81 (#MA5-13548, Thermo Fisher Scientific) or anti-GFP (#sc-9996, Santa Cruz Biotechnology) mouse monoclonal Abs, or anti-Syntenin rabbit polyclonal Ab (#ab19903, Abcam) for 60 min at RT. After three washing steps of 10 min each with PBS containing 0.1% Tween 20, membranes were incubated with AlexaFluor488-conjugated goat anti-mouse (#A11017) or anti-rabbit (#A11070) IgG, both from Thermo Fisher Scientific, for 30 min at RT. Finally, membranes were washed thrice (10 min each) in PBS containing 0.1% Tween 20, rinsed in deionized H_2_O, and antigen-Ab complexes were visualized using the iBright FL1000 system (Thermo Fisher Scientific).

### Immunocytochemistry

Cells grown on glass-bottom dishes coated with poly-D-lysine (#P35GC-1.5-14-C, MatTek Corporation) were processed for immunocytochemistry. They were fixed in 4% paraformaldehyde (PFA) for 15 min, permeabilized with 0.2% Tween 20 for 15 min, and blocked with 1% BSA for 1 h at RT. Actin was labeled with AlexaFluor647-conjugated phalloidin (#A22287, Thermo Fisher Scientific) for 60 min, then cells were counterstained with DAPI (#D9542, Sigma-Aldrich) for 30 min at RT. Cells were imaged in PBS using the Nanoimager S Mark II microscope (Oxford Nanoimaging (ONI)) with 100X oil-immersion objective.

### Stochastic optical reconstruction microscopy

EVs were immunolabeled and imaged using the EV Profiler Kit (#EV-MAN-1.0, ONI) by employing direct stochastic optical reconstruction microscopy (dSTORM) technique. Briefly, approximately 1.0 × 10^7^ EVs were immobilized on microfluidic chips, fixed with F1 solution (provided in the kit) for 10 min, and permeabilized with increasing concentrations of Triton X-100 at 0.001, 0.01, or 0.1% for 10 min. EVs were then incubated for 50 min with fluorescently labeled Abs diluted in permeabilization buffer or PBS for non-permeabilized immunolabeling. The following antibodies were used, either provided in the kit or purchased from a different vendor: CD9-CF488 (kit, excitation (ex)/emission (em): 490/515 nm), CD63-CF568 (kit, ex/em: 562/583 nm), CD81-CF647 (kit, ex/em: 650/665 nm), Syntenin-AlexaFluor488 (kit, ex/em: 490/525 nm), Alix-AlexaFluor488 (#NB100-65678AF488, ex/em: 490/525 nm, Novus Biologicals), Spike-AlexaFluor647 (#51-6490-82, ex/em: 650/671 nm, Thermo Fisher Scientific), Nucleocapsid-AlexaFluor488 (#ab283243, ex/em: 495/519 nm, Abcam), and GFP-AlexaFluor647 (#A-31852, ex/em: 650/671 nm, Thermo Fisher Scientific). Finally, samples were again fixed with F1 for 10 min, and freshly prepared dSTORM-imaging buffer was added prior to image acquisition. Using the Nanoimager S Mark II microscope (ONI) with 100X oil-immersion objective, labeled proteins were imaged sequentially at 25, 35, and 50% power for the 640, 561, and 488 nm lasers, respectively, at 1,000 frames per channel with the angle of illumination set to 52.5°. Prior to the start of the imaging session, channel mapping was calibrated using 0.1 µm TetraSpeck beads (#T7279, Thermo Fisher Scientific). Data were processed on NimOS software (version 1.19, ONI). Subpopulation analyses of EVs that express one, two, or three markers were analyzed using ONI’s online platform called CODI (https://alto.codi.bio). Herein, we performed a density-based clustering analysis with drift correction and filtering to evaluate each vesicle.

### Statistical analysis

All experiments were carried out at least in triplicate. The error bars in the graphical data represent the mean ± standard deviation (S.D.) as indicated in the figure legends. Two-tailed Student’s *t*-test was used to determine statistical significance. *P* values less than 0.05 were considered significant. All graphs were created using GraphPad Prism 9.

## RESULTS

**Figure 1:**
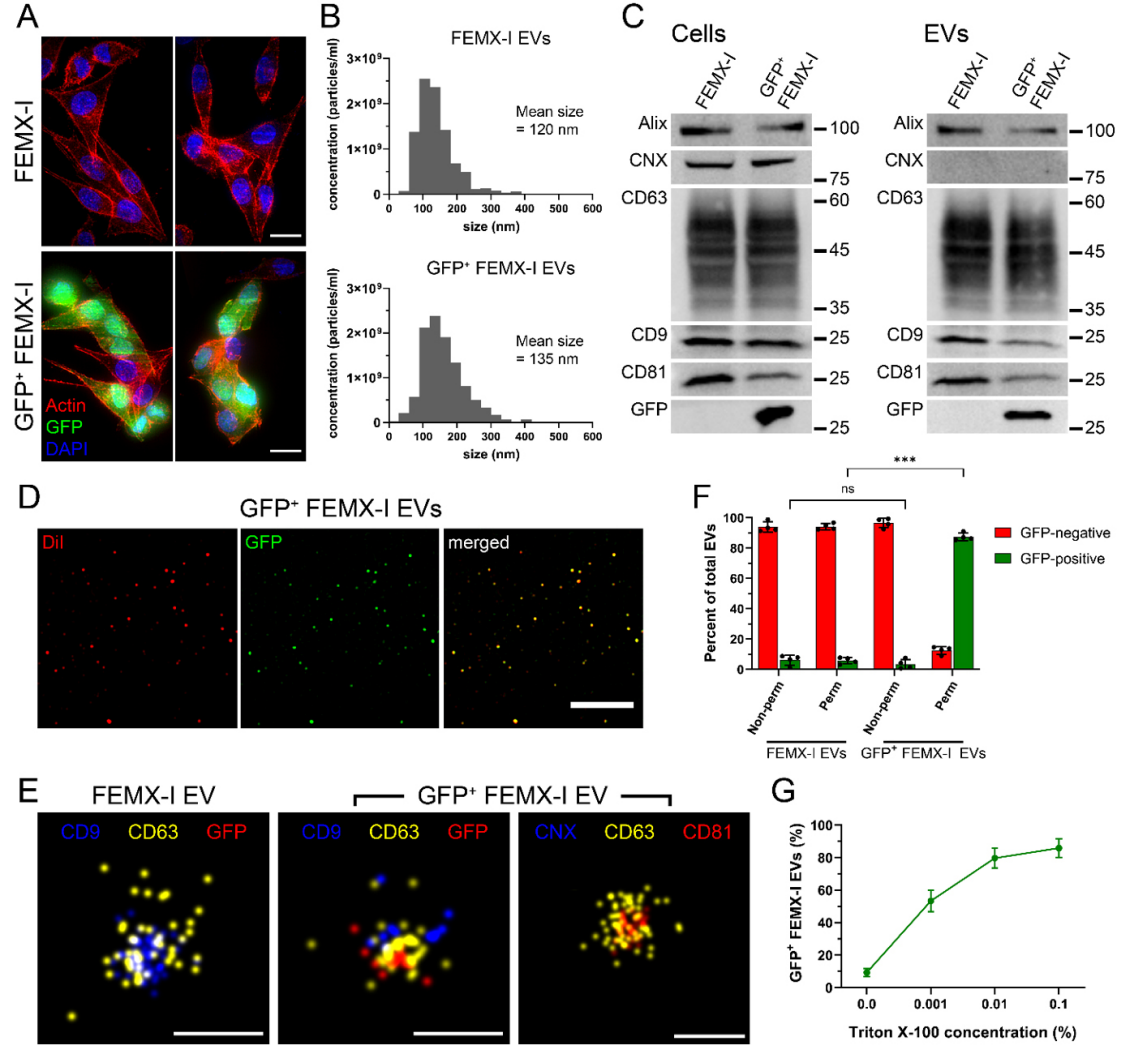
Intravesicular protein detection by dSTORM. (A) Confocal micrographs of FEMX-I and GFP^+^ FEMX-I cells stained with phalloidin (actin, red) and DAPI (blue). (B) Size and concentration of EVs derived from cells in panel A were determined by NTA. (C) Cells and EVs were solubilized and subjected to Western blot analysis. Note the absence of GFP in the parental cells and EVs and of calnexin (CNX) in both EV samples. (D) EVs derived from GFP^+^ FEMX-I were labeled with DiI dye and analyzed by confocal microscopy. (E and F) EVs were permeabilized and labeled for CD9 or CNX (blue), CD63 (yellow), and GFP or CD81 (red) and analyzed by dSTORM then quantified. (G) EVs derived from GFP^+^ FEMX-I were subjected to increasing concentrations of Triton X-100, analyzed by dSTORM, and quantified. For all EV quantification, a range of 2,000 to 3,000 EV particles were analyzed per replicate per condition. N.s.: not significant; ***: *P* < 0.001; Scale bar: 10 µm (A, D) or 100 nm (E).

To establish a method for the detection of intravesicular proteins, we stably expressed the green fluorescent protein (GFP) in human FEMX-I melanoma cells by transfecting them with a plasmid carrying the GFP gene [Figure 1A]. Subsequently, we followed a previously published protocol to isolate extracellular vesicles (EVs) from both parental and GFP-expressing FEMX-I cells^[30]^. As determined by NTA, both EV populations exhibited similar size and concentration [Figure 1B]. They were further characterized by immunoblotting for the presence of classical EV markers, namely CD9, CD81, CD63, and Alix, as well as for the absence of calnexin (CNX) [Figure 1C]. To verify GFP expression, blots were also probed using anti-GFP antibodies. Note that both parental cell lysates and EVs were negative for GFP signal [Figure 1C]. We then investigated whether all EVs released from GFP-positive cells carried GFP in them. To this end, all EVs were stained with a lipophilic membrane dye, DiI, and then analyzed by confocal microscopy [Figure 1D]. More than 95% of EVs were positive for both GFP and DiI.

We used dSTORM to determine the spatial localization of single molecules relative to the EV compartment. Accordingly, we immobilized EVs from parental and GFP-positive FEMX-I cells on PEG-biotin-coated microfluidic chips. Next, EVs were permeabilized using 0.01% Triton X-100, then immunolabeled using anti-CD9, -CD63, and -GFP antibodies [Figure 1E and F]. Note that an anti-GFP antibody conjugated to appropriate fluorescent dyes was used because GFP itself would not fluoresce in dSTORM imaging due to its inability to attain the “blinking” event necessary for generating single-molecule images. As expected, we did not detect GFP signal in the parental EV population. In contrast, upon permeabilization, GFP was observed in EVs isolated from GFP-positive FEMX-I cells. These EVs were additionally immunolabeled for calnexin, a negative marker of EVs, and CD81, another marker of EVs [Figure 1E]. To determine the percentage of EVs harboring GFP in them, density-based cluster analysis was utilized. Herein, the percentage of EVs positive for the respective marker was assessed. EVs that were permeabilized and isolated from GFP-positive cells showed positive GFP signals in about 90% of the total EV population per sample compared to the non-permeabilized condition or those isolated from parental cells [Figure 1F].

Due to their size, EVs are susceptible to destruction when exposed to high concentrations of detergent. Thus, a permeabilization curve was performed to determine the optimal concentration wherein GFP signals in EVs were detected at near maximum [Figure 1G]. It is important to note that increasing Triton X-100 concentration up to 0.01% resulted in a proportional increase in the observable GFP signal. Above this concentration, no significant differences were noted in terms of intensity enhancement and distribution of GFP across the EV population [Figure 1G]. Moreover, NTA analysis did not show any significant change in the size and concentration of EVs upon exposure to the highest concentration of Triton X-100 used in this study, i.e., 0.1% (data not shown).

**Figure 2:**
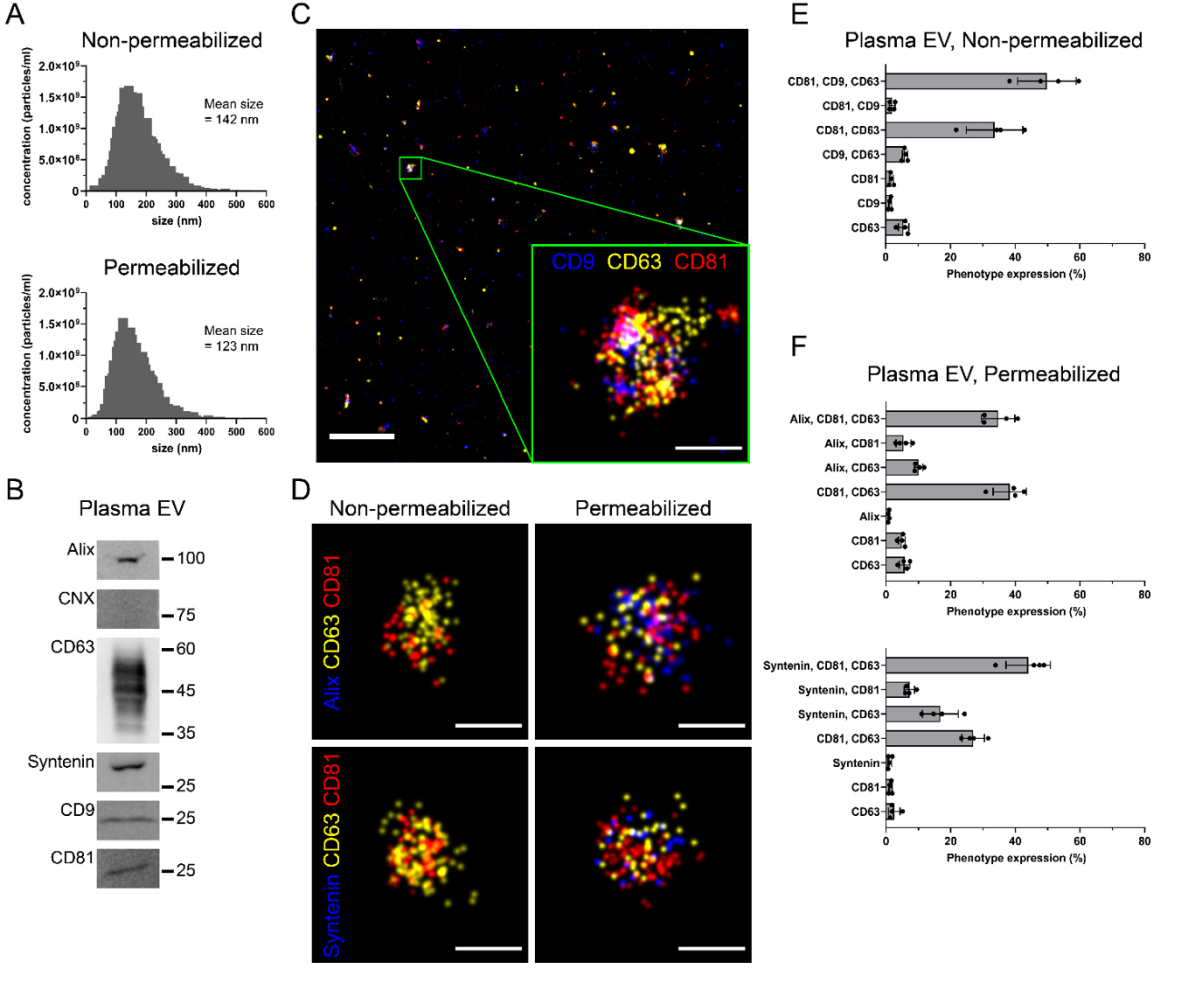
EVs isolated from the blood plasma of healthy volunteers. (A) Size and concentration of non-permeabilized and permeabilized EVs purified from peripheral blood were determined by NTA; (B and C) Plasma EVs were characterized by immunoblotting (B) for the indicated proteins and dSTORM imaging (C) for CD9 (blue), CD63 (yellow), and CD81 (red). A magnified view of a single EV is presented (C); (D) Non-permeabilized and permeabilized EVs were immunolabeled for Alix or Syntenin (blue), CD63 (yellow), and CD81 (red) and analyzed by dSTORM; (E and F) EVs expressing single-, double-, and triple-positive of specific protein markers as indicated were quantified. For all EV quantification, a range of 2,000 to 3,000 EV particles were analyzed per replicate per condition. Scale bars, 10 µm (C, overview) or 100 nm (C, magnified, D).

To extend the applicability of our method, we analyzed EVs isolated from the blood plasma of healthy individuals in our study. The procedure involved subjecting EDTA-containing blood to sequential centrifugation steps, and the resultant EVs were quantified by NTA as above. Subsequently, permeabilization of the EV samples was carried out, followed by the re-evaluation of concentration and size distribution [Figure 2A]. These observations collectively underscored the absence of any discernible impact from permeabilization on the size and concentration of EVs [Figure 2A]. As anticipated, immunoblotting confirmed that these EVs were positive for CD9, CD63, CD81, Syntenin, and Alix and negative for CNX [Figure 2B]. By dSTORM, we discerned distinct triple-positive (CD9^+^, CD63^+^, and CD81^+^) populations of EVs [Figure 2C].

Subsequently, we assessed the sensitivity of the method to detect endogenous intravesicular markers, particularly Alix and Syntenin. Upon permeabilization or not, EVs were immunolabeled for CD63, CD81, and Alix or Syntenin [Figure 2D-F]. Both Alix and Syntenin were observed only in the permeabilized sample, as expected by their known intravesicular localization [Figure 2D]. Using cluster analysis, the distribution of triple-, double-, and single-positive markers was measured. Herein, more than 50% of the EVs demonstrated triple positivity for the tetraspanins CD81, CD9, and CD63, while more than 35% exhibited double positivity for CD81 and CD63 [Figure 2E]. These data suggest that the majority of blood EVs originated from multi-vesicular bodies inside cells due to high expression of CD63, and thus are considered the exosome type of EVs, which is in line with other studies that describe the spatial distinction of CD9- and CD63-positive EV biogenesis^[31]^. Other combinations of single and double positive EV surface markers represented a minor fraction of the total EV population. Following permeabilization, Alix and Syntenin were detected and quantified by cluster analysis [Figure 2F]. Notably, the subpopulations of triple-positive Alix^+^CD81^+^CD63^+^ EVs and Syntenin^+^CD81^+^CD63^+^ EVs accounted for ~35 and ~45% of the total EV population, respectively. In contrast, the double-positive combination of Syntenin and CD63 constituted nearly 20% of the EV population, while the population of Alix^+^CD63^+^ EVs was less than 10% of the total EV population, suggesting that more exosomes contain Syntenin than Alix. Whether these findings physiologically display a functional effect is yet to be determined.

We then investigated whether we could apply our technique to detect viral proteins of SARS-CoV-2 inside EVs isolated from the blood plasma of individuals who previously tested positive for COVID-19. Previous studies have shown that EVs carry the spike and nucleocapsid protein components of the virus^[22,23]^. However, these studies were limited to immunoblotting detection, wherein the protein source comes from solubilized materials. Improper purification of blood EVs may also lead to inaccurate detection as soluble forms of viral proteins in blood are indistinguishable in current protein assay preparations (i.e., immunoblotting). By using dSTORM analysis, we were able to determine the relative location of these proteins within the EV compartment.

**Figure 3:**
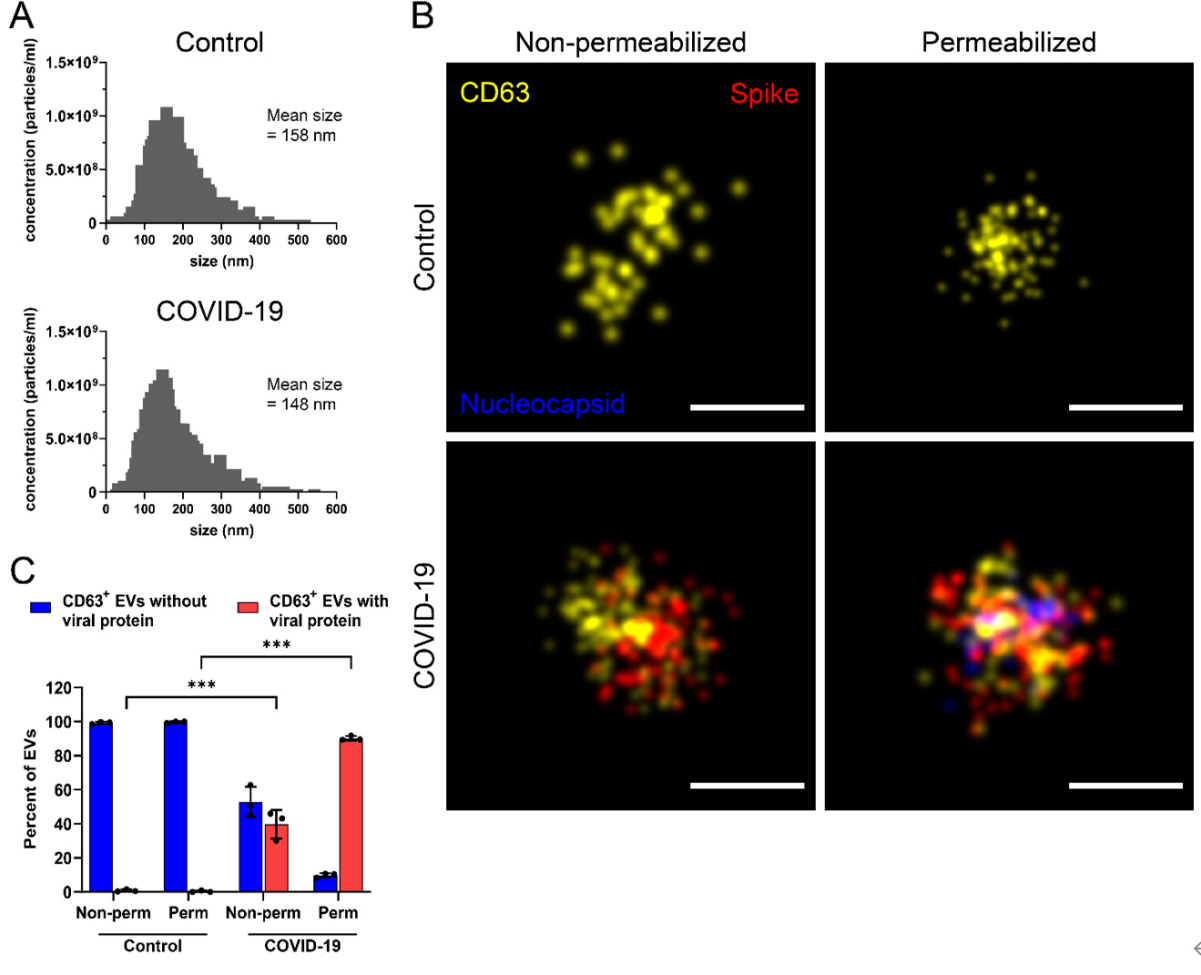
Comparison of EVs isolated from blood plasma of healthy and Sars-CoV2-infected volunteers. (A) Size and concentration of EVs purified from peripheral blood of healthy and COVID^+^ patients were determined by NTA; (B and C) Non-permeabilized and permeabilized EVs were immunolabeled for nucleocapsid (blue), CD63 (yellow), and spike (red) and analyzed by dSTORM (B), and then quantified (C); For all EV quantification, a range of 2,000 to 3,000 EV particles were analyzed per replicate per condition. ***: *P* < 0.001; Scale bars: 100 nm (B).

EVs isolated from healthy control and COVID-19-positive individuals were characterized by NTA, with both preparations having similar size and concentration [Figure 3A], which is in line with other studies^[32,33]^. EVs were then immobilized on a microfluidic chip, and non-permeabilized and permeabilized samples were immunolabeled for CD63, spike, and nucleocapsid [Figure 3B and C]. In the control population, spike and nucleocapsid were absent regardless of permeabilization condition. In EVs from COVID-19-positive individuals, spike protein was detected in non-permeabilized samples, suggesting that it is membrane-bound. Nucleocapsid, in contrast, was observed only upon permeabilization [Figure 3B]. Cluster analysis showed that, after permeabilization, about 90% of CD63^+^ EVs contained viral proteins, which may be used to correlate disease progression of infected or recovering individuals [Figure 3C]. Interestingly, the amount of detected spike proteins also increased in the permeabilized EVs of COVID-19-positive samples, suggesting that some spike proteins are sequestered inside EVs, which may be important in the modulation of the immune response over time, for example, in progression to long COVID-19 symptoms [Figure 3B and C]. A similar study described a distinct population of Spike protein inside vesicles upon exposure to proteinase K, which destroyed Spike on the membrane surface^[34]^.

## DISCUSSION

Here, we have successfully demonstrated that the cargo of EVs can be analyzed at the single EV level without disrupting their physical characteristics. Many reports have associated EVs with a number of diseases, including viral infections, cancer, cardiovascular disease, autoimmune disorders, and neurological diseases, where they may play a role in development and progression^[35]^. These reports have generated a growing interest in the potential of blood plasma EVs for diagnostic and prognostic applications. EV cargo can provide information about the cell of origin, the state of the cell, and the biological processes involved in the disease.

SARS-CoV-2 spike is a major target of the immune system, particularly inducing the production of neutralizing antibodies (nAbs) by infection or vaccines. Interestingly, spike-carrying EVs may stimulate humoral and cellular immune responses to SARS-CoV-2 and promote the persistence of high-titer nAbs in blood plasma^[36]^. At the same time, spike-carrying EVs released from spike-expressing cells may serve as decoys for anti-spike nAbs, promoting viral infection^[22]^. EVs are also reported to carry and transfer viral RNA to other cell types, which can further spread and exacerbate symptoms of COVID-19^[34]^. EVs have also been reported to contain complete SARS-CoV-2 virions^[37]^. Thus, in COVID-19 patients and other viral diseases, EVs may induce immunity or act as bait for nAbs, in part because EVs from infected cells can transfer viral proteins to uninfected cells and to immune cells in order to mask the infection or trigger a response^[21]^. In particular, the discovery of the intricate relationship between SARS-CoV-2, EVs, and the immune system that occurs in COVID-19 patients has important implications for disease progression, prevention, and therapy of COVID-19. In this regard, analysis of both surface and intravesicular protein cargo of patients infected with SARS-CoV-2 at the single EV level without disrupting the physical characteristics of EVs may have very significant clinical potential. The method we have developed is accurate and rapid while maintaining the physical characteristics of EVs, which is crucial for utilizing them as reliable diagnostic and prognostic markers.

We tested our protocol in EVs isolated from cultures of GFP-expressing cells and validated the protocol in bloodborne EVs isolated from healthy and COVID-19 infected individuals. GFP proteins are not natively expressed in mammalian cells but are packaged inside EVs of GFP-expressing cells. Thus, they make a great tool in detecting EV cargo upon permeabilization followed by immunolabeling for GFP, when comparing EVs isolated from parental and GFP-positive cells. By a dose-response investigation, we have successfully identified the optimal Triton-X 100 concentration that provides the best permeabilization/maximum signal without destroying or disrupting the surface composition of EVs. We confirmed the lack of effect of the permeabilization process on the size and composition of EVs using NTA and antibody labeling of surface markers and subsequent analysis by super-resolution microscopy. We further confirmed the expression of GFP in the EVs of these cells by immunoblotting. Detection of Alix and lack of staining for calnexin confirmed permeabilization. In bloodborne EVs, we were able to detect the intravesicular EV proteins Syntenin and Alix only in the permeabilized EV population. Additionally, our detergent concentration did not affect the structural integrity of bloodborne EVs, as evidenced by their NTA profile.

Furthermore, we investigated the presence and density of viral proteins in SARS-CoV-2-derived EVs isolated from COVID-19 patients by comparing them to plasma EVs isolated from healthy individuals and collected prior to 2018 (i.e., pre-COVID-19 time). Because of our novel protocol that allowed us to measure the EV cargo while maintaining their shape and surface composition, we identified for the first time an equal amount of spike proteins on the surface and inside EVs against the common understanding that EVs carry spike proteins on their surface^[36]^. The additional viral protein load could further increase the risk of prolonged infection by immune system activation through T cells and DC interaction. This could pose a serious risk of excessive inflammatory response that would not have been expected with routine surface spike analysis.

We would like to acknowledge certain limitations of some of the employed techniques. First, the concentration of our permeabilization buffer may need to be optimized for EVs isolated from other cell lines. Also, because of the limited number of subjects studied, the percentage of markers expressed in EVs cannot be generalized. Lastly, in this study we only used ultracentrifugation to isolate EVs, and we cannot exclude that other EV preparation techniques, such as density gradient ultracentrifugation and size-exclusion chromatography, affect permeabilization efficiency and integrity of the surface of EVs.

## DECLARATIONS

### Availability of data and materials

Data and materials supporting the published data will be shared on reasonable requests.

### Copyright

© The Author(s) 2023.

## References

[1] Raposo G, Stoorvogel W (2013). Extracellular vesicles: exosomes, microvesicles, and friends. J Cell Biol.

[2] Santos MF, Rappa G, Karbanová J (2023). HIV-1-induced nuclear invaginations mediated by VAP-A, ORP3, and Rab7 complex explain infection of activated T cells. Nat Commun.

[3] Santos MF, Rappa G, Karbanová J, Kurth T, Corbeil D, Lorico A (2018). VAMP-associated protein-A and oxysterol-binding protein-related protein 3 promote the entry of late endosomes into the nucleoplasmic reticulum. J Biol Chem.

[4] Gould SJ, Booth AM, Hildreth JE (2003). The Trojan exosome hypothesis. Proc Natl Acad Sci U S A.

[5] Kalluri R, LeBleu VS (2020). The biology, function, and biomedical applications of exosomes. Science.

[6] Green TM, Santos MF, Barsky SH, Rappa G, Lorico A (2016). Analogies between cancer-derived extracellular vesicles and enveloped viruses with an emphasis on human breast cancer. Curr Pathobiol Rep.

[7] Nolte-'t Hoen E, Cremer T, Gallo RC, Margolis LB (2016). Extracellular vesicles and viruses: are they close relatives?. Proc Natl Acad Sci U S A.

[8] Lenassi M, Cagney G, Liao M (2010). HIV Nef is secreted in exosomes and triggers apoptosis in bystander CD4+ T cells. Traffic.

[9] Chen L, Feng Z, Yue H (2018). Exosomes derived from HIV-1-infected cells promote growth and progression of cancer via HIV TAR RNA. Nat Commun.

[10] Arakelyan A, Fitzgerald W, Zicari S, Vanpouille C, Margolis L (2017). Extracellular vesicles carry HIV Env and facilitate Hiv infection of human lymphoid tissue. Sci Rep.

[11] Testa JS, Apcher GS, Comber JD, Eisenlohr LC (2010). Exosome-driven antigen transfer for MHC class II presentation facilitated by the receptor binding activity of influenza hemagglutinin. J Immunol.

[12] Zicari S, Arakelyan A, Palomino RAÑ (2018). Human cytomegalovirus-infected cells release extracellular vesicles that carry viral surface proteins. Virology.

[13] Izquierdo-Useros N, Naranjo-Gómez M, Archer J (2009). Capture and transfer of HIV-1 particles by mature dendritic cells converges with the exosome-dissemination pathway. Blood.

[14] Robbins PD, Morelli AE (2014). Regulation of immune responses by extracellular vesicles. Nat Rev Immunol.

[15] Vincent-Schneider H, Stumptner-Cuvelette P, Lankar D (2002). Exosomes bearing HLA-DR1 molecules need dendritic cells to efficiently stimulate specific T cells. Int Immunol.

[16] Utsugi-Kobukai S, Fujimaki H, Hotta C, Nakazawa M, Minami M (2003). MHC class I-mediated exogenous antigen presentation by exosomes secreted from immature and mature bone marrow derived dendritic cells. Immunol Lett.

[17] Théry C, Duban L, Segura E, Véron P, Lantz O, Amigorena S (2002). Indirect activation of naïve CD4+ T cells by dendritic cell-derived exosomes. Nat Immunol.

[18] Fu C, Peng P, Loschko J (2020). Plasmacytoid dendritic cells cross-prime naive CD8 T cells by transferring antigen to conventional dendritic cells through exosomes. Proc Natl Acad Sci U S A.

[19] Ye Y, Gaugler B, Mohty M, Malard F (2020). Plasmacytoid dendritic cell biology and its role in immune-mediated diseases. Clin Transl Immunology.

[20] Marcoux G, Laroche A, Hasse S (2021). Platelet EVs contain an active proteasome involved in protein processing for antigen presentation via MHC-I molecules. Blood.

[21] Pesce E, Manfrini N, Cordiglieri C (2021). Exosomes recovered from the plasma of COVID-19 patients expose SARS-CoV-2 spike-derived fragments and contribute to the adaptive immune response. Front Immunol.

[22] Troyer Z, Alhusaini N, Tabler CO (2021). Extracellular vesicles carry SARS-CoV-2 spike protein and serve as decoys for neutralizing antibodies. J Extracell Vesicles.

[23] DeMarino C, Lee MH, Cowen M (2023). Detection of SARS-CoV-2 nucleocapsid and microvascular disease in the brain: a case report. Neurology.

[24] Chu H, Chan JF, Yuen TT (2020). Comparative tropism, replication kinetics, and cell damage profiling of SARS-CoV-2 and SARS-CoV with implications for clinical manifestations, transmissibility, and laboratory studies of COVID-19: an observational study. Lancet Microbe.

[25] Mondal A, Ashiq KA, Phulpagar P, Singh DK, Shiras A (2019). Effective visualization and easy tracking of extracellular vesicles in glioma cells. Biol Proced Online.

[26] Hartjes TA, Mytnyk S, Jenster GW, van Steijn V, van Royen ME (2019). Extracellular vesicle quantification and characterization: common methods and emerging approaches. Bioengineering.

[27] Rappa G, Santos MF, Green TM (2017). Nuclear transport of cancer extracellular vesicle-derived biomaterials through nuclear envelope invagination-associated late endosomes. Oncotarget.

[28] Rappa G, Mercapide J, Anzanello F, Pope RM, Lorico A (2013). Biochemical and biological characterization of exosomes containing prominin-1/CD133. Mol Cancer.

[29] Théry C, Witwer KW, Aikawa E (2018). Minimal information for studies of extracellular vesicles 2018 (MISEV2018): a position statement of the international society for extracellular vesicles and update of the MISEV2014 guidelines. J Extracell Vesicles.

[30] Santos MF, Rappa G, Karbanová J (2021). Itraconazole inhibits nuclear delivery of extracellular vesicle cargo by disrupting the entry of late endosomes into the nucleoplasmic reticulum. J Extracell Vesicles.

[31] White ED, Walker ND, Yi H, Dinner AR, Scherer NF, Rosner MR (2022). Volumetric microscopy of CD9 and CD63 reveals distinct subpopulations and novel structures of extracellular vesicles in situ in triple negative breast cancer cells. BioRxiv.

[32] Mao K, Tan Q, Ma Y (2021). Proteomics of extracellular vesicles in plasma reveals the characteristics and residual traces of COVID-19 patients without underlying diseases after 3 months of recovery. Cell Death Dis.

[33] Yim KHW, Borgoni S, Chahwan R (2022). Serum extracellular vesicles profiling is associated with COVID-19 progression and immune responses. J Extracell Biol.

[34] Craddock V, Mahajan A, Spikes L (2023). Persistent circulation of soluble and extracellular vesicle-linked Spike protein in individuals with postacute sequelae of COVID-19. J Med Virol.

[35] Cheng L, Hill AF (2022). Therapeutically harnessing extracellular vesicles. Nat Rev Drug Discov.

[36] Pontelli MC, Castro ÍA, Martins RB (2022). SARS-CoV-2 productively infects primary human immune system cells in vitro and in COVID-19 patients. J Mol Cell Biol.

[37] Xia B, Pan X, Luo RH (2023). Extracellular vesicles mediate antibody-resistant transmission of SARS-CoV-2. Cell Discov.

